# The Effects of Propranolol and Corticosterone on Susceptibility Priming in a Mouse Model of Social Defeat Stress

**DOI:** 10.21203/rs.3.rs-8369302/v1

**Published:** 2025-12-23

**Authors:** Alessia Manganaro, Jack Yin Zhang, Giulia Zanni, Clementine Fillinger, Dani Dumitriu

**Affiliations:** 1 Center for Early Relational Health, Columbia University, New York, NY, United States.; 2 Division for Child and Adolescent Health, Department of Pediatrics, Columbia University Irving Medical Center, New York, NY, United States.; 3 Division of Developmental Neuroscience, Department of Psychiatry, Columbia University Irving Medical Center, New York, NY, United States.; 4 Departments of Neuroscience and Neurosurgery, Icahn School of Medicine at Mount Sinai, New York, NY, United States.; 5 Research Foundation for Mental Hygiene, New York State Psychiatric Institute, NY, New York, United States.; 6 University de Strasbourg, Strasbourg, France.

## Abstract

Animal models for stress-related mood disorders aim to mirror the diverse spectrum of stress responses observed in humans, which ultimately determine an individual’s vulnerability or capacity to cope effectively with the stressor. The acute social defeat stress (ASDS) protocol allows a rapid phenotypic classification of mice subjected to social stress to interrogate the early neural patterns beyond divergent stress outcomes. Although ASDS alone doesn’t cause chronic depression-like behaviors, it “primes” the mouse to be highly susceptible to a subsequent, subthreshold stress (StSDS). Here, we tested whether pharmacological manipulation shortly before ASDS can counter this susceptibility priming. Specifically, we examined the β-adrenergic receptor antagonist propranolol and the glucocorticoid corticosterone. Male mice received acute intraperitoneal injections of propranolol, corticosterone, or saline prior to ASDS, followed one week later by StSDS and social interaction testing. Propranolol partially prevented the emergence of social avoidance following subsequent StSDS, without modifying the behavioral outcome of ASDS. In contrast, corticosterone administered at different doses, failed to prevent susceptibility priming to later StSDS, disrupting also the typical acute ASDS behavioral profiling at low and medium doses. The results suggest that blocking β-adrenergic signaling during an acute stressor can reduce future stress susceptibility, while underscoring the complex and dose-dependent effects of glucocorticoids on stress responsivity.

## Introduction

Chronic stressors are more likely to lead to psychopathology than single stressful events [1]. In humans, cumulative stress has been shown to increase the prevalence of several physical and mental health conditions, including hypertension, substance abuse disorders, and depression in susceptible individuals [1,2,3]. Evidence suggests that exposure to an initial stressor potentiates risk of subsequently developing psychopathology after additional stressors by “priming” stress circuits. This has been dubbed the two-hit hypothesis [4]. It suggests that complex diseases such as schizophrenia and depression are the result of two or more major disruptions during critical periods — the first increasing the risk of pathogenesis, while the second triggering actual disease onset. Both disturbances are necessary to induce the disorder, but neither alone is sufficient [4,5].

A possible mechanism by which this priming effect manifests is the process of memory consolidation [6,7,8]. Whereas the complex mechanisms by which stress impacts memory have not yet been fully characterized, growing evidence supports an important role played by stress hormones and their targets [6,9,10], which are being investigated in the treatment of post-traumatic stress disorder (PTSD) both in animal models and humans [11,12,13]. One compound targeting the stress response pathway is the beta-adrenergic receptor antagonist, propranolol. Across multiple rodent studies, propranolol administration diminished fear response following reactivation of the learned fear memory [14,15,16]. Comparable effects have been demonstrated in humans as well, with Vaiva et al. reporting lower rates of PTSD in patients prescribed propranolol post-trauma compared to those who did not [17].

A second compound of interest is cortisol in humans, or its counterpart, corticosterone, in rodents. Several studies reported patients with PTSD to have lower cortisol levels [12,19]. A single high-dose corticosteroid injection in the acute aftermath of a traumatic event has gained some traction as a post-exposure prophylaxis to supplement a diminished stress response [11,13]. In rats exposed to a predator scent, high-dose corticosterone treatment reduced the prevalence of extreme behavioral disruption 30 days later [18]. A similar impairment in fear memory was reported in mice infused with corticosterone following conditioning with electric foot shock [20]. These data make propranolol and corticosterone candidates to study the inhibition of susceptibility priming by an initial stressful event on a future repeat stressor.

To investigate the potential of propranolol and corticosterone in preventing susceptibility priming to social stress, we utilized the social defeat stress (SDS) paradigm, a commonly used animal model of stress-induced depressive-like behavior [21]. During SDS, the experimental animal undergoes bouts of physical aggression from a larger, aggressive conspecific. Following SDS, a large proportion of mice exhibit physiological and social deficits, including alterations to various neurotransmitter systems, metabolic disturbances, and social avoidance [21]. In the present study, two variants of SDS were used: acute social defeat stress (ASDS) and subthreshold social defeat stress model (StSDS). The ASDS model, established in our lab to investigate pre-existing neurocircuitry differences underlying individual variability in stress responses, induces quantifiable differences in social avoidance between control and defeat animals at one-hour post-defeat, without inducing long-term pervasive depressive-like behaviors [22]. The StSDS model usually fails to induce significant differences in social avoidance in defeated animals [21,23]. However, we previously observed that mice subjected to StSDS after having first experienced ASDS are primed to exhibit social avoidance and a susceptible phenotype [22]. Here, we investigated whether acute administration of either propranolol or corticosterone, preceding an acute stress priming event (ASDS), is capable of mitigating or inhibiting susceptibility priming to a subsequent subthreshold stressor (StSDS) in adult male mice.

## Materials and methods

### Animals

8-week-old male C57BL/6 J mice (Jackson Laboratories, Bar Harbor, ME), and retired male breeder CD-1 mice (Charles River Laboratory, Wilmington, MA) were used in the experiments. All mice were housed in a temperature-controlled room operating under 12-hour diurnal conditions (light phase: 08:00–20:00 EST). Mice were group-housed four per cage in standard microisolator cages with wood-chip bedding. Food and water were provided ad libitum. Animal identification was maintained via tail labeling with permanent marker. Any animal that exhibited visibly reduced locomotion and/or open wounds exceeding 1 cm in diameter resulting from defeat was immediately removed from the study and euthanized.

The male CD-1 mice were ordered as retired breeders under four months of age, and single-housed in a room separate from the experimental mice. The aggressor animals were screened before the experiment, according to the protocol detailed by Golden et al [21]. CD-1 mice who fail to meet aggression criteria were used as the novel, non-aggressive social target during the social interaction test.

All animals used in this study were maintained in accordance with the recommendations posed by the National Institutes of Health (NIH) Guide for the Care and Use of Laboratory Animals. All experiments were performed in strict adherence with the guidelines established by the Institutional Animal Care and Use Committee at the Icahn School of Medicine at Mount Sinai and conducted in compliance with NIH animal care guidelines.

### Drugs and preparation

All injections were performed intraperitoneally (IP). Across the two experiments, three compounds were administered. Normal saline (SAL) was used as the control injection. Propranolol hydrochloride (West-Ward Pharmaceuticals, cat. 0143-9872-10) (PROP) was administered at a dose of 10mg/kg. Both the SAL and PROP solutions were administered at room temperature.

The corticosterone (CORT) solution was prepared from ≥98.5% powdered corticosterone (Sigma-Aldrich, Cat. 50-22-6), ethanol, and corn oil. We used three doses: 0.1mg/kg (low), 5mg/kg (medium), and 25mg/kg (high). In each, the appropriate amount of CORT was prepared by first suspending the powder in 0.5 mL of ethanol. This suspension was then vortexed and sonicated at 37 degrees Celsius, until the solute was mostly dissolved. 4.5 mL of corn oil was then added to the mixture, vortexed, and sonicated, until complete dissolution was achieved. These solutions were prepared fresh the day of the experiment, and they were kept in the sonicator/water bath at 37 degrees Celsius between injections to maintain solution integrity. The drug/vehicle solutions were injected at the same time of day in a volume of 1ml/kg body weight.

### Social defeat stress (SDS)

**Figure 1:**
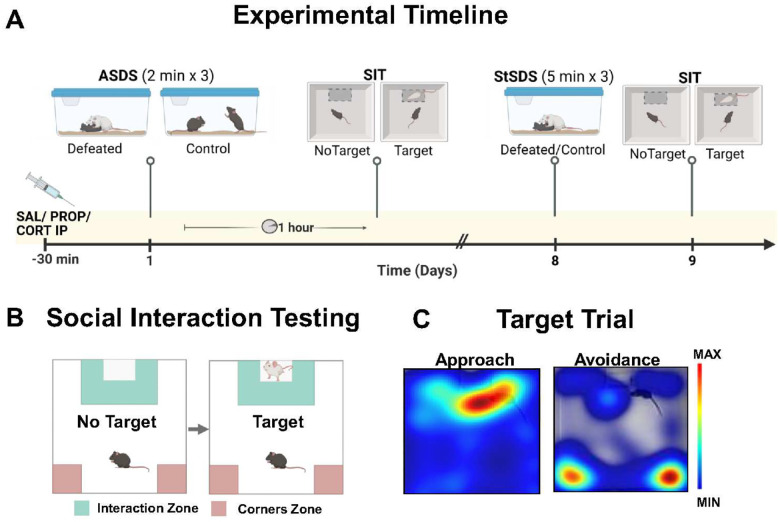
Experimental design of the Propranolol and Corticosterone experiments. (**A**) Experimental Timeline (**B**) Schematic representation of the two consecutive trials of SIT, without and with target (non-aggressive CD1) in the interactor. The “interaction zone” and the “corners zone” are depicted in green and red respectively. (**C**). Examples of mean occupancy heatmaps for two mice showing approaching and social avoidant behavior respectively during the SIT Target session.

Behavioral testing was conducted between 12:00 and 16:00. Animals were subjected to ASDS, followed by StSDS one week later (Fig.1A). Both protocols are adaptations of the resident-intruder paradigm described by Golden et al [21] and previously published by our lab [22]. The ASDS protocol consisted of three consecutive rounds of two-minute defeat sessions with three different CD-1 aggressors, followed by 54 minutes of single housing in a clean cage prior to social interaction (SI) testing. In the StSDS, animals similarly underwent three rounds of defeat with different aggressors, with each round lasting five minutes, with 15-minute rest intervals housed in their home cages between rounds. Following the third defeat round, StSDS mice were then returned to their home cages and 24 hours later tested in SI. The ASDS control group underwent a controlled social interaction instead of defeat. Non-aggressive conspecifics were presented for three encounters. Mice with a history of ASDS underwent defeat sessions in the subsequent StSDS. The injection control group that did not undergo ASDS, non-aggressive conspecifics were used as the control group in the StSDS paradigm.

### Social interaction test (SIT)

Following ASDS and StSDS, SI was tested identically at one hour and 24 hours respectively, under red light conditions as previously described [21]. SI quantifies social preference versus avoidance (Fig. 1C). Experimental mice were placed into an opaque Plexiglas open-field arena (42×42×42 cm) with a rectangular wire-mesh enclosure placed against one of the inner walls. Experimental animals were first allowed to explore the arena for 150 sec with “no target” present in the enclosure for habituation and baseline activity monitoring (Fig.1A&1B). The animals were then removed, the arena cleaned with 70% ethanol, and a novel non-aggressive CD1 “target” mouse placed into the enclosure to serve as the social target. Experimental animals were then returned to the arena for another 150 sec.

Video-tracking (EthoVision XT 10 software, Noldus Information Technology) was used to record animal location and behavior. Total time spent in the “interaction zone”, an unmarked rectangular region around the interaction enclosure (14×24 cm); and “corner zone” (9×9 cm, located on the opposite wall from the enclosure, cumulative of the two corners) were quantified. Interaction and corner ratios were calculated by dividing time spent in the zones during “target” present by time during “no target”. SI scores were calculated as the ratio of the total time the experimental animal spent in the interaction zone when the social target was present, to the total time spent there when the target was absent. (Fig.1B &1C) [23, 21, 22].

### Exclusionary criteria

During SDS, mice undergo multiple physical attacks by the aggressive CD1. Animals excessively wounded during ASDS were excluded from the experiment and from analysis (Table S1). Freezing behavior occurs in response to stress- or fear-inducing stimuli. In the context of the SI test following ASDS/StSDS, a freezing phenotype is uncommon [22]. When it occurs, it may result in a misleading SI score and phenotype assignment, especially if the mouse freezes in the interaction zone. To address this, we established a mean velocity threshold of ≥1.8 cm/s during the SIT, such that all animals below this minimum value were labeled as “freezers” and not classified as susceptible or resilient. In total, 12 animals were excluded following this criterion (Table S1& Fig.S1).

### Exp. 1: Effect of propranolol on susceptibility priming

The experiment was conducted over the course of nine days (Fig.1A), with four groups of randomly assigned animals: animals injected with saline and subjected to controlled social interactions with conspecifics (SAL CTR, n=12); animals injected with saline and subjected to ASDS (SAL DEF, n=19); animals injected with propranolol and subjected to controlled social interactions with conspecifics (PROP CTR, n=12); and animals injected with propranolol and subjected to ASDS (PROP DEF, n=16). Of the 72 experimental animals, one mouse was excluded and euthanized due to excessive wounding (Table S1). In total 56 animals were considered in the final analysis across timepoints.

On the first day, animals underwent ASDS, followed by SI one hour later (Fig.1A). One hour before ASDS, animals were removed from their group-housed home cage and placed into clean individual cages. Animals were then injected with either saline or propranolol according to body weight. Thirty minutes post-injection, defeat group animals underwent ASDS (SAL DEF; PROP DEF), while control group animals underwent controlled social interactions (SAL CTR; PROP CTR). Following SI testing, animals returned to their individual cages and group-housed with their prior cage mates.

StSDS occurred one week later (Fig.1A). All animals underwent subthreshold defeat in the absence of drug or saline, regardless of group assignment during the ASDS. Following the conclusion of this micro-defeat, animals returned to their original home cage and housing room. SIT was repeated 24 hours after the conclusion of StSDS. Animals were weighed before this final SI.

### Exp. 2: Effect of corticosterone on susceptibility priming

Animals in this experiment underwent the same experimental design as those in Exp. 1 (Fig.1A). 40 animals were randomly assigned to six different groups: (i) animals receiving the lowest dose of corticosterone (0.1mg/kg) and acute control interaction (CI) with conspecifics (LOW CORT AS-C, n=6); (ii) animals receiving the lowest dose of corticosterone and ASDS (LOW CORT AS-D, n=6); (iii) animals receiving the medium dose of corticosterone (5mg/kg) and acute CI with conspecifics (MED CORT AS-C, n=7); (iv) animals receiving the medium dose of corticosterone and ASDS (MED CORT AS-D, n=7); (v) animals receiving the highest dose of corticosterone (25mg/kg) and acute CI with conspecifics (HIGH CORT AS-C, n=7); and (vi) animals receiving the highest dose of corticosterone and ASDS (HIGH CORT AS-D, n=7).

All animals underwent defeat in the StSDS paradigm one week following ASDS, regardless of group assignment during the acute phase (Fig.1A). No animal in this experiment met exclusion criteria for either freezing behavior or wound severity (Table S1).

### Statistical analysis

We used repeated mixed effect model and two-way repeated measures ANOVA, followed by a Šídák’s or Tukey’s post-hoc multiple comparisons test as recommended, to compare the difference between each pair of group means (CTR vs DEF, and SAL vs PROP/CORT) and within pair (ASDS vs StSDS). Statistical significance was defined as p<0.05. All statistical analyses were conducted in Graphpad Prism 10.

## Results

### Propranolol reduces ASDS-induced stress-priming to subsequent StSDS.

**Figure 2:**
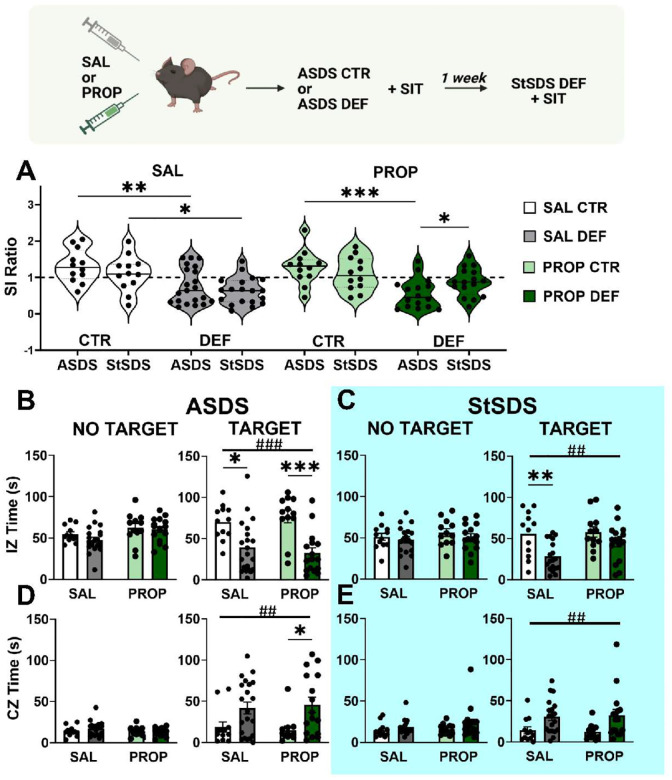
Propranolol administration reduces ASDS-induced stress priming to StSDS. Experimental timeline of the propranolol experiment on top. **(A)** Violin dot plot graph showing SI ratios in control (CTR) and defeated (DEF) groups following ASDS and StSDS in saline (SAL) and propranolol (PROP) treatment groups. Dashed line denotes the threshold for social avoidance (SI ratio = 1). (**B**) Bar graph of the absolute time animals spent in the interaction zone (IZ) during SI after ASDS, showing no difference between groups when the CD1 target is not present (left panel) and in the presence of the CD1 target (right panel). (**C**) Bar graph of the time the animal spent exploring the IZ during SIT after StSDS in absence (left) and in presence (right) of the CD1 target. (**D**) Bar graph of the time the animal spent exploring the corners zone (CZ) during SIT after ASDS. (**E**) Bar graph of the time the animal spent exploring the CZ during SI after StSDS. Data are presented as mean +/− SEM. Main effect # P < 0.05, ## P < 0.001, ### P < 0.0001. Post-hoc: *P < 0.05, **P < 0.01, *** P < 0.001.

We previously showed that ASDS, robustly induce social avoidance on SI one-hour post-defeat [22]. Although not resulting in persistent social avoidance, ASDS primes to subsequent social stress, including StSDS paradigm that would not otherwise lead to susceptibility under control conditions [22]. We hypothesized that this priming is mediated by consolidation of the initial stress memory and that disrupting consolidation immediately after ASDS would prevent priming to subsequent stressor. To test whether propranolol blocks ASDS-induced stress-priming, mice underwent ASDS in the presence or absence of propranolol (PROP vs SAL), followed by StSDS one week later. Mice received propranolol or saline injections 30 minutes before ASDS and tested for social avoidance versus social preference using standard SI testing one hour after initiation of ASDS (Fig.1A&2A). Because here we were interested in the effects of the animal’s history (stress-priming and/or drug treatment) on subsequent susceptibility, all mice, irrespective of drug treatment, further underwent StSDS (i.e, no controlled social interaction with conspecifics was conducted), and no drug or saline was administered at time of StSDS.

Using a repeated mixed-effect model to evaluate SI ratios across time (ASDS vs StSDS) and treatment groups (SAL CTR, SAL DEF, PROP CTR, PROP DEF), we looked at the effect of ASDS and treatment (SAL vs PROP) on SI ratios. We found a significant main effect of behavior (CTR vs DEF) on the SI ratios (F_3,57_=10.15, *p<0.0001*), and a significant interaction between SI ratios across time and treatment (F_3,55_=3.039, *p=0.0366*).

Consistent with previous ASDS results, multiple comparison analysis revealed that saline treated defeated animals had significantly decreased SI ratio compared to controls (SAL CTR _ASDS_ vs SAL DEF _ASDS_, *p=0.0042*) (Fig.2A). Similarly, propranolol defeated animals showed significantly decreased SI ratio compared to controls (PROP CTR _ASDS_ vs PROP DEF _ASDS_, *p=0.0002*) (Fig.2A). These data prove that the ASDS paradigm induced robust social avoidance in all defeated animals irrespective of treatment.

We next examined the effects of propranolol treatment on ASDS-induced stress-priming to subsequent StSDS. We confirmed that saline animals with a history of ASDS showed the stress-priming effect in the subsequent StSDS (SAL CTR _StSDS_ vs SAL DEF _StSDS_, *p=0.0261*) (Fig.2A). However, after StSDS no differences emerged between ASDS control and defeated animals in the propranolol-treated groups (PROP CTR _StSDS_ vs PROP DEF _StSDS_, *p=0.6846*), indicating that propranolol blunted the susceptibility-priming effect of ASDS (Fig.2A). On the contrary, the decreased SI ratio observed during ASDS (PROP DEF _ASDS_) amounting to 0.55 was completely rescued in the propranolol defeated (PROP DEF _StSDS_) amounting to 0.86 during StSDS (*p=0.0262*).

We further looked at the effects of ASDS on the temporal dynamics of SI behavior by examining the time spent in the interaction zone (Fig.2B&2C) and the corner zone (Fig.2D&2E) with or without the novel CD-1 mouse target. No effect of ASDS or treatment was seen on interaction zone and corner zone times in the NO-target phase. We found a significant main effect of ASDS both in the interaction zone time (F_1,57_=27.87, *p<0.0001*) and the corner zone time (F_1,57_=11.85, *p=0.0011*) but no main effect of propranolol treatment nor interaction when the target was present. Post-hoc analysis showed that defeated animals spent significantly less time in the interaction zone both in CTR DEF (*p=0.0051*) and PROP DEF (*p=0.0001*), as opposed to significant more time in the corner zone only in the PROP DEF group (*p=0.0181*). These results demonstrate that propranolol injection before ASDS does not affect ASDS-induced social avoidance observed one-hour post-defeat. Similarly, we looked at how StSDS impacts primed and unprimed dynamics of SI behavior by examining the time spent in the interaction zone (Fig.2F&2G) and the corner zone (Fig.2H&2I) with or without the novel CD-1 mouse target. No effect of StSDS or treatment was seen on interaction zone and corner zone times in the NO-target phase. We found a significant main effect of StSDS both in the interaction zone time (F_1,55_=11.62, *p=0.0012*) and the corner zone time (F_1,55_=11.00, *p=0.0016*) but no main effect of propranolol treatment nor interaction when the target was present. Post-hoc analysis showed that only CTR DEF animals spent significantly less time in the interaction zone (*p=0.0041*), but no difference was observed in the PROP DEF group. This confirms that defeated animals display significant changes in basic movement or zone occupancy patterns during both ASDS and subsequent StSDS, supporting this stress priming effect, which was blunted by propranolol.

Interestingly, we noted an increased number of “freezer” animals in the propranolol-treated defeat group (Table S1 & Sup.Fig.1), an effect not observed in the propranolol control group, suggesting that propranolol may amplify freezing selectively in the context of recent social stress, thus representing an adaptive response to the recent social stressor and propranolol treatment.

Together, these data demonstrate that although propranolol does not alter the immediate behavioral consequences of ASDS, it selectively disrupts the consolidation of susceptibility priming, preventing the emergence of social avoidance during the later StSDS exposure.

### Corticosterone affect immediate ASDS-outcome has no effects on susceptibility-priming.

**Figure 3:**
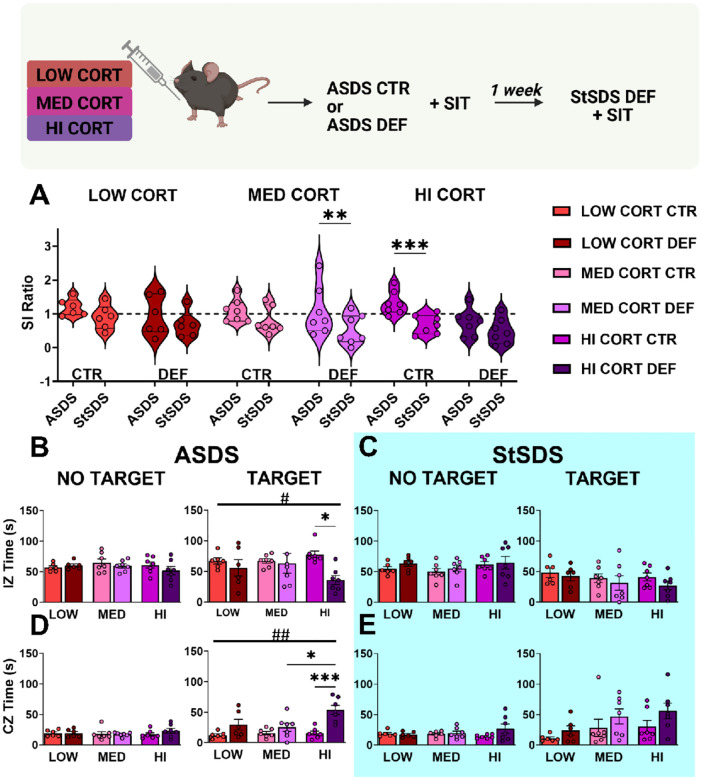
Corticosterone administration interferes with ASDS social avoidance. Experimental timeline of the corticosterone experiment on top. **(A)** Violin dot plot graph showing SI ratios in control (CTR) and defeated (DEF) groups following ASDS and StSDS in the three mice groups injected, low dose (LOW CORT), medium dose (MED CORT) and high dose (HI CORT) of corticosterone. Dashed line denotes the threshold for social avoidance (SI ratio = 1). (**B**) Bar graph of the absolute time animals spent in the interaction zone (IZ) during SIT following ASDS, in the no target and target sessions. (**C**) Bar graph of the absolute time animals spent in IZ during SI after StSDS without the CD1 target (left panel) and in the presence of the CD1 target (right panel). (**D**) Bar graph of the time the animals spent exploring the corner zone (CZ) during SI after ASDS, in the no target and target sessions. (**E**) Bar graph of the time the animal spent exploring the CZ during SI following StSDS without CD1 target and with CD1 target. Data are presented as mean +/− SEM. Main effect # P < 0.05, ## P < 0.001, ### P < 0.0001. Post-hoc: *P < 0.05, **P < 0.01, *** P < 0.001.

In the second experiment, we evaluated whether different doses of corticosterone, low, medium and high, injected 30 minutes before ASDS blocked the susceptibility-priming to subsequent StSDS (Fig.3A). The two-way repeated-measures ANOVA conducted to evaluate SI ratios across time (ASDS vs StSDS) and corticosterone dose (LOW, MED, HI), revealed a strong main effect of the timepoint on SI ratio (F_1,34_=29.87, *p<0.0001*), indicating that animals tested after StSDS exhibited significantly lower SI ratios than during ASDS across conditions. In contrast, there was no main effect of corticosterone dose (F_2,54_=1.264, p=0.3021) and no significant interaction overall between timepoint and dose (F_2,54_=0.9976, p=0.4340). In particular, the multiple comparisons analysis revealed that mice receiving MED dose of CORT showed lower SI ratios following StSDS (MED DEF_ASDS_ vs MED DEF_StSDS_, *p=0.0020*). Similarly, ASDS control mice treated with HI dose of CORT exhibited lower SI ratios following StSDS (HI CTR_ASDS_ vs HI CTR_StSDS_, *p=0.0006*). These results demonstrate that corticosterone treatment at any dose did not reduce susceptibility-priming as hypothesized. Notably, we did not observe significant differences in SI ratios after ASDS between control and defeated mice at any corticosterone dose, in contrast to the standard ASDS-induced social avoidance (lower SI ratios) in male mice (Fig.3A). This suggests that corticosterone injections blunted the expression of social avoidance after social acute stress. This effect was apparent at low, medium, and high doses, with no corticosterone condition showing defeat-induced reduction in SI ratio after ASDS.

To evaluate the behavioral patterns observed during SI following ASDS, we looked at the absolute time spent in the different zones of the arena during the no target and target trials. Two-way repeated-measures ANOVA detected an overall stress effect during ASDS on absolute time in the interaction zone (F_1,34_=5.718, *p=0.0225*), and absolute time in the corner zone (F_1,34_=21.11, *p<0.0001*) in presence of the CD1 target mouse (respectively, Fig.3C&3E). Only for the HI CORT, the multiple comparison analysis revealed a significant reduction in IZ time (F_1,34_=5.718, p=0.0225), and a significant increase in CZ time (F_1,34_=21.11, p<0.0001) in presence of the CD1 target mouse (Fig.3C&3E, respectively). Because these effects were main effects of defeat, they were observed across all corticosterone conditions and were not altered by drug treatment. This indicate that ASDS still induced detectable patterns of social avoidance, even though no represented in the SI ratios.

Although no social avoidance was detected immediately after ASDS in the medium corticosterone group (MED CORT DEF), the significant drop in SI ratio reported following StSDS for this group indicates that susceptibility-priming still occurred. Additionally, the significant decrease in SI ratio after StSDS of HIGH CORT control animals suggests that high-dose corticosterone administered during a non-aggressive interaction may itself function as a priming stimulus.

In summary, corticosterone treatment (i) disrupts the normal acute social avoidance response to ASDS, (ii) fails to prevent susceptibility priming, and (iii) may even promote priming at certain doses.

## Discussion

The susceptibility priming effect is a phenomenon by which an animal exposed to an acute stressor is predisposed towards greater susceptibility following exposure to a subsequent stressor. This priming effect occurs even when the second stressor is mild and does not induce significant susceptibility in animals under normal conditions, as is the case of StSDS. Our interest lies in the initial response and neuro-circuitry state underlying individual variation in stress-induced phenotype, thus we investigated the effect of two promising therapeutic compounds for the treatment of PTSD on the susceptibility-priming induced by acute stressor: the beta-adrenergic antagonist, propranolol, and the glucocorticoid, corticosterone.

Here, we investigated the capacity of a single acute administration of either propranolol, or corticosterone, to inhibit the susceptibility priming effect. We previously reported that ASDS in adult mice is sufficient to induce susceptibility to a mild stressor, such as StSDS [22]. We confirmed this phenotype by showing that the saline group, which experienced ASDS defeat, exhibited increase social avoidance, as indicated by reduced SI scores, decreased total time spent in the interaction zone and increased total time spent in corner zone in presence of the CD1. Consistently with the ASDS paradigm, defeated animals displayed lower SI ratios than control animals, in both saline and propranolol groups. This suggests that administration of propranolol had no effect on the initial ASDS, making it an optimal compound candidate, to mitigate the neuro-circuitry changes associated with susceptibility priming after the initial stress response has occurred. Interestingly, we showed that social avoidance post-StSDS is reduced by propranolol injection 30 minutes prior to the onset of ASDS, which resulted in significant mitigation of susceptibility-priming. Our data demonstrated that propranolol administration prevented animals from developing further social susceptibility-priming to StSDS, as indicated by no significant differences in the time spent in the interaction zone and corner zone with or without the novel CD-1 mouse target in the propranolol group. Our results are consistent with multiple line of evidence showing that propranolol reduces stress-related behavioral responses. In chronic social defeat, chronic unpredictable stress, and restraint stress models, systemic propranolol attenuates anxiety-like and anhedonia-like behaviors, normalizes stress-induced hyperarousal, and mitigates neuroinflammatory and noradrenergic adaptations linked to vulnerability [24, 25, 26]. In classic Pavlovian fear-conditioning paradigms, propranolol administered before or shortly after stress exposure disrupts consolidation of aversive associations, reliably impairing fear memory reactivation, resulting in diminished freezing and reduced amygdala-dependent fear expression [15, 27]. Beyond amygdala circuits, recent work indicates that propranolol also modulates hippocampal network activity during fear retrieval, including reduced neural activation within the hilus of the dentate gyrus, suggesting broader effects on mnemonic processes that contribute to stress-related memory expression [28].

In addition, we noticed that some animals excluded from the main analysis, presented a higher level of freezing during the initial ASDS encounter. In this group, we observed a distinct pattern of response to propranolol. In saline-treated mice, high level of freezing was reflected in heightened social avoidance after StSDS, consistent with the idea that pronounced passive coping during the initial trauma predicts greater susceptibility priming. However, in the propranolol group, freezer animals did not go on to display the expected exacerbation of social avoidance in the StSDS. Instead, propranolol-treated freezers showed preserved interaction zone exploration and did not exhibit the pronounced reduction in SI ratio observed in saline freezers. Consistent with prior work, propranolol effectively mitigates fear-related freezing and weakens reconsolidation of aversive memories in classical fear-conditioning paradigms (e.g., tone or contextual fear conditioning) [29, 30, 31, 32]. Yet to our knowledge, this is the first study examining the role of propranolol on social memory. The novel aspect of our finding is that unlike prior studies which focused merely on “freezing” or passive fear responses, our work highlights the social component of stress vulnerability, namely social avoidance and social exploration, showing that propranolol can prevent the priming of social susceptibility following repeated mild stress. More importantly, propranolol prevents the maladaptive consolidation of stress-related memory that was otherwise observed in saline freezer animals. Thus, β-adrenergic blockade appears not only to mitigate susceptibility priming at the group level but also to specifically buffer the exacerbated phenotype observed in highly vulnerable individuals and prevent the emergence of a high-risk phenotype after re-exposure to a mild social challenge.

These findings demonstrate that β-adrenergic blockade influences both acute stress reactivity and long-term plasticity, with efficacy observed across multiple dosing schedules and stress paradigms. However, several key questions remain. In our study, we looked only at males, and literature data on sex differences are limited. In addition, multiple dose and timing parameters in acute and chronic stress paradigm should be tested. Addressing these issues through validated animal models is essential for reducing the current translational gap of propranolol use in clinical trials.

Contrary to the propranolol results, corticosterone administered prior to ASDS did not block susceptibility-priming, regardless of dose. Instead, at medium and high doses, corticosterone might have enhanced vulnerability to stress, as evidenced by significant SI ratio decrease in both MED CORT DEF and HI CORT CTR groups when comparing ASDS and StSDS. Importantly, the outcomes of the acute defeat phase of the corticosterone study deviated from those predicted by the paradigm itself. Specifically, whereas there was a main effect of the social stressor on the absolute time spent in the interaction zone and the corners zone in the target trial, only mice that received the high dose of corticosterone spent significant less time in proximity of the CD1 and more in the corners compared to the controls. This suggests that the corticosterone injection had some impact on the acute defeat, as the paradigm normally would induce a clear distinction between the mean SI scores of controls and defeated animals. One possible reason for the mild behavioral interference of the compound in this initial phase is the injection time in our experimental design. The decision to inject 30 minutes prior to defeat rather than immediately post-defeat was due to concerns regarding the bioavailability of the compound suspended in oil, necessary to supplement the stress-induced corticosterone release. However, the dose-dependent results in our study might suggests that CORT was absorbed in the system at the time of early testing, and rapid negative feedback of the HPA axis occurred as a result of plasma glucocorticoid concentrations rising to levels exceeding the range of physiological homeostasis. According to Andrews et al. [33], an increase in glucocorticoid receptor (GR) activation leads to dose-dependent suppression of basal HPA axis activity in female rats. The effect lasts up to several hours but is subject to a finite dose-dependent delay, with the earliest time suppression onset 40 min after low dose steroid injection. This delay could explain why both low and medium doses of CORT prior to the acute stressor failed to induce a robust social avoidance following ASDS. This assumes that the onset of the HPA axis suppression preceded the SIT, possibly amplifying the stress activation during defeat, while buffering the stress response during SI testing. The high dose of 25 mg/Kg of the steroid compound used here might have shifted the onset of the above suppression after SIT, resulting in disrupted social interaction pattern in the defeated group, as indicated by the increased corner time and decreased time spent in the interaction zone. Moreover, this framework would justify the augmented susceptibility towards the second stressor for all the groups, assuming that the injection of CORT interfered with the timing of glucocorticoid peak detected downstream the HPA axis, without necessarily abolishing fear memory acquisition and possible consolidation in critical brain regions like the Amygdala. Indeed, it has been shown that glucocorticoid increases in response to stress enhance stress-related memories [25, 26, 34]. It’ s important to mention that the effects of corticosterone follow an inverted-U curve across dose and timing, determined by differential engagement of mineralocorticoid receptors (MRs) (high affinity) vs glucocorticoid receptors (GRs) [35, 36, 37, 38, 39]. This effect also known as hormesis [40] is specific to the level of expression of MRs and GRs in the brain areas sensitive to CORT levels, with the Para Ventricular Nucleus and Amygdala having stronger GR-driven excitability/plasticity changes than Hippocampus. Hence, it is plausible that the injection of corticosterone before ASDS acted at a different time scale for each dose in activated brain areas, still producing a long-term effect on subsequent stressors. Thus, a medium or high dose of corticosterone was sufficient to prime animals towards a second stressor, as observed by a decreased SI ratio in the high corticosterone control group. In agreement with Jaszczyk et. Al. [41], acute-to-subacute CORT elevation can produce real but modest and selective effects with a broad time-course: some changes are rapid (within hours), others delayed and some persist after hormone returns to baseline. Consistently, in our study the presence of CORT in all groups may have masked the normal time course of endogenous corticosterone following an acute stressor. Further study with different time administration windows and multiples readout may offer a meaningful insight into the mechanisms of corticosterone time of action and its role in the susceptibility priming. Experiments comparing pre- vs post-stress CORT levels will help explain better the current results. As for propranolol, future experiments should include females as most of the present studies for preclinical model on CORT protective effects have been performed mainly on males.

Overall, this study extends our knowledge of the impact of an initial stressful event on a future re-exposure to stressors, and it opens the possibility for acting on and interfering with this priming effect. Additionally, the promising effect of propranolol will allow us to compare its effects on the pre-existing and unmodified neurobiological mechanisms responsible for individual variability in the stress response.

## Supplementary Files

This is a list of supplementary files associated with this preprint. Click to download.

- supplementarytable.png
- supplementaryFig.png

**Table S1: Animal Table**. Number of mice labelled as freezers or wounded per group and experiment.

**Figure S1: Propranolol increases freezing behavior after ASDS but is absent after StSDS. (A)** Comparisons of SI ratios after ASDS and StSDS for saline and propranolol defeated injected mice categorized as freezers during SIT following ASDS. Paired t-test reported. **(B)** Bar graphs of the absolute time in the IZ during no target and target sessions of SIT after ASDS for control, defeated and freezer mice in the saline and propranolol condition. One way ANOVA with post-hoc Tukey. **(C)** Bar graphs of the absolute time in the IZ during no target and target sessions of SIT after StSDS for the same groups in **B.** One way ANOVA with post-hoc Tukey. Data are presented as mean +/− SEM. Post-hoc: *P < 0.05, **P < 0.01, *** P < 0.001.
